# Barnidipine compared to lercanidipine in addition to losartan on endothelial damage and oxidative stress parameters in patients with hypertension and type 2 diabetes mellitus

**DOI:** 10.1186/s12872-016-0237-z

**Published:** 2016-04-12

**Authors:** Giuseppe Derosa, Amedeo Mugellini, Rosa Maria Pesce, Angela D’Angelo, Pamela Maffioli

**Affiliations:** Department of Internal Medicine and Therapeutics, University of Pavia, Fondazione IRCCS Policlinico San Matteo, Pavia, P.le C. Golgi, 2, 27100 Pavia, Italy; Center for the Study of Endocrine-Metabolic Pathophysiology and Clinical Research, University of Pavia, Pavia, Italy; Molecular Medicine Laboratory, University of Pavia, Pavia, Italy; PhD School in Experimental Medicine, University of Pavia, Pavia, Italy

**Keywords:** Barnidipine, Losartan, Oxidative stress

## Abstract

**Background:**

Essential hypertension has been extensively reported to cause endothelial dysfunction. The aim of this study was to evaluate the effects of barnidipine or lercanidipine, in addition to losartan, on some parameters indicative of endothelial damage and oxidative stress in hypertensive, type 2 diabetic patients.

**Methods:**

One hundred and fifty one patients were randomised to barnidipine, 20 mg/day, or lercanidipine, 20 mg/day, both in addition to losartan, 100 mg/day, for 6 months. We assessed BP every month, in addition, patients underwent ambulatory blood pressure monitoring (ABPM). We also assessed: fasting plasma glucose (FPG), glycated hemoglobin (HbA_1c_), some markers such as high-sensitivity C-reactive protein (Hs-CRP), tumor necrosis factor-α (TNF-α), metalloproteinase-2 (MMP-2) and -9 (MMP-9), soluble vascular adhesion protein-1 (sVCAM-1), soluble intercellular adhesion protein-1 (sICAM-1), isoprostanes and paraoxonase-1 (PON-1).

**Results:**

Both barnidipine and lercanidipine resulted in a significant reduction in blood pressure, even if the reduction obtained with barnidipine + losartan was greater than that obtained with lercanidipine + losartan. Data recorded with ABPM also showed a similar trend. Barnidipine + losartan reduced the levels of Hs-CRP, TNF-α, sVCAM-1, sICAM-1, and isoprostanes both compared to baseline and to lercanidipine + losartan.

**Conclusions:**

Barnidipine + losartan gave an improvement of some parameters indicative of endothelial damage and oxidative stress in diabetic and hypertensive patients.

**Trial registration:**

NCT02064218, ClinicalTrials.gov

## Background

Essential hypertension has been extensively reported to cause endothelial dysfunction, characterised by unbalanced vasodilation and vasoconstriction, increased oxidative stress, vascular inflammation, alteration of prothrombotic and fibrinolytic pathways, abnormal smooth muscle cell proliferation, and impaired repair mechanisms [1]. In particular, metalloproteinases (MMPs) have been implicated as a cardiovascular risk factor [2], and a large body of evidence asserts the role of MMPs in atherosclerosis [3]. It is well known that the main cause of acute coronary syndromes is the plaque disruption with subsequent superimposed intracoronary thrombus leading to prolonged coronary obstruction [4]. Both MMP-2 and MMP-9 are synthesized and secreted locally in atherosclerotic lesions, predominantly by monocyte derived macrophages and endothelial cells [5]. It has been already reported that circulating MMPs levels are elevated in hypertensive patients [6], in obese subjects [7], in patients with acute coronary syndrome [8], with type 2 diabetes mellitus [9] or with combined dyslipidemia [10]. According to latest European Cardiology Society guidelines, combination of anti-hypertensive therapy provides greater blood pressure lowering effects than single-agent therapy, and the added benefit of an angiotensin receptor blocker (ARB) and dihydropyridine calcium channel blocker (CCB) combination is a minor incidence of adverse events [11]. Angiotensin receptor blocker and CCB combination also proved to have some pleiotropic effects such as improvement of insulin sensitivity [12], and prevention of new episodes of atrial fibrillation in hypertensive diabetic patients [13].

However, not all CCBs have the same properties, and studies comparing CCBs effects on inflammations are lacking. Among CCBs, barnidipine and lercanidipine proved to be the ones with longer lasting effect.

In particular barnidipine hydrochloride differs from other CCBs, such as nifedipine or nisoldipine, in its water solubility. In vitro studies in rats have revealed that barnidipine metabolism is catalyzed by liver microsomes, specifically by cytochrome P450. The pharmacological effects of barnidipine are longer lasting than those of other CCBs such as nifedipine; sustained occupancy of dihydropyridine receptors by barnidipine was observed in vitro as well as in vivo [14].

Lercanidipine, instead, is a lipophilic, dihydropyridine calcium antagonist, and its high membrane partition coefficient provides a long-lasting effect at receptor and membrane levels, allowing for once daily administration. Its slow onset of action helps to avoid reflex tachycardia associated with other dihydropyridines [15].

The aim of this study was to evaluate the effects of barnidipine or lercanidipine, in addition to losartan, on some parameters indicative of endothelial damage and oxidative stress in patients with hypertension and type 2 diabetes mellitus.

## Methods

### Study design

This randomised, double-blind, controlled study was conducted at the Department of Internal Medicine and Therapeutics, IRCCS Policlinico San Matteo, PAVIA, Italy.

The study protocol was conducted in accordance with the Declaration of Helsinki and its amendments, and the Good Clinical Practice Guidelines. It was approved by the IRCCS Policlinico San Matteo Ethical Committee and all patients provided written informed consent prior to entering the study. TRIAL REGISTRATION: *ClinicalTrials.gov* NCT02064218.

### Patients

We enrolled 151 hypertensive patients with mild to moderate hypertension, type 2 diabetes mellitus, normocholesterolemic [low density lipoprotein cholesterol (LDL-C) < 160 mg/dl], overweight outpatients, aged ≥ 18 of either sex (Table 1).

**Table 1:** Baseline characteristics of patients in the two treatment groups

	Losartan+barnidipine	Losartan+lercanidipine
N	75	76
Age (years)	60.5 ± 8.9	60.7 ± 8.8
Sex (male/female)	37/38	36/40
BMI (Kg/m^2^)	28.5 ± 1.3	28.2 ± 1.1
Duration of diabetes (months)	9.3 ± 7.2	9.1 ± 7.1
Duration of hypertension (months)	6.4 ± 3.6	6.3 ± 3.5
eGFR (ml/min/1.73 m^2^)	84.1 ± 8.5	82.5 ± 7.6

Data are means ± SD

*BMI* body mass index, *eGFR* estimated glomerular filtration rate

Patients were evaluated for eligibility according to the following inclusion criteria:

- systolic blood pressure (SBP) ≥ 140 mmHg < 180 mmHg and/or diastolic blood pressure (DBP) ≥ 90 mmHg < 105 mmHg
- in therapy with losartan
- well controlled type 2 diabetes mellitus (HbA_1c_ ≤ 7.5 %)

The exclusion criteria were secondary hypertension, severe hypertension (SBP ≥ 180 mmHg or DBP ≥ 105 mmHg), hypertrophic cardiomyopathies due to etiologies other than hypertension, history of heart failure, history of angina, stroke, transient ischemic cerebral attack, coronary artery bypass surgery or myocardial infarction any time prior to visit 1, concurrent known symptomatic arrhythmia, liver dysfunction (AST or ALT values exceeding 2-fold the upper limit), creatinine > 1.5 mg/dl, known hypersensitivity to the study drugs. Pregnant women as well as women of childbearing potential were excluded.

Suitable subjects, identified from review of case notes and/or computerized clinic registers were contacted personally or by telephone. No changes in anti-diabetic treatment happened during the study.

### Treatments

The patients fulfilling the inclusion and exclusion criteria, were randomised to lercanidipine 20 mg/day, or barnidipine, 20 mg/day, in addition to losartan 100 mg/day for 6 months. Both lercanidipine, and barnidipine were supplied as identical, opaque, white capsules in coded bottles to ensure the blind status of the study. Randomisation was done using a drawing of envelopes containing randomisation codes prepared by a statistician. A copy of the code was provided only to the responsible person performing the statistical analysis. The code was only broken after database lock, but could have been broken for individual subjects in cases of an emergency. Medication compliance was assessed by counting the number of pills returned at the time of specified clinic visits. At baseline, we weighed participants and gave them a bottle containing a supply of the study medication for at least 100 days. Throughout the study, we instructed patients to take their first dose of new medication on the day after they were given the study medication. At the same time, all unused medication was retrieved for inventory. All medications were provided free of charge.

### Diet and exercise

Patients were already following a controlled-energy diet (near 600 Kcal daily deficit) based on American Heart Association (AHA) recommendations [16] that included 50 % of calories from carbohydrates, 30 % from fat (6 % saturated), and 20 % from proteins, with a maximum cholesterol content of 300 mg/day and 35 g/day of fibre. Patients were not treated with vitamins or mineral preparations during the study.

Standard diet advice was given by a dietitian and/or specialist doctor. Dietitian and/or specialist doctor periodically provided instruction on dietary intake recording procedures as part of a behaviour modification program and then later used the subject’s food diaries for counselling. Individuals were also encouraged to increase their physical activity by walking briskly for 20 to 30 min, 3 to 5 times per week, or by cycling. The recommended changes in physical activity throughout the study were not assessed.

### Assessments

Before starting the study, all patients underwent an initial screening assessment that included a medical history, physical examination, vital signs, and a 12-lead electrocardiogram. We assessed blood pressure (BP) every month, in addition, patients underwent ambulatory blood pressure monitoring (ABPM), at baseline and at the end of the study. We also collected blood sample to assess: fasting plasma glucose (FPG), glycated hemoglobin (HbA_1c_), some markers indicative of endothelial damage such as high-sensitivity C-reactive protein (Hs-CRP), tumor necrosis factor-α (TNF-α), metalloproteinase-2 (MMP-2) and -9 (MMP-9), soluble vascular adhesion protein-1 (sVCAM-1), soluble intercellular adhesion protein-1 (sICAM-1). We also evaluated some markers of oxidative stress such as isoprostanes and paraoxonase-1 (PON-1).

All plasmatic parameters were determined after a 12-h overnight fast. Venous blood samples were taken for all patients between 08.00 and 09.00 A.M. We used plasma obtained by addition of Na_2_-EDTA, 1 mg/ml, and centrifuged at 3000 g for 15 min at 4 °C. Immediately after centrifugation, the plasma samples were frozen and stored at -80 °C for no more than 3 months. All measurements were performed in a central laboratory.

Blood pressure measurements were obtained from each patient (left arm) in the sitting position by physicians blinded to treatment using a standard mercury sphygmomanometer (Erkameter 3000; ERKA, Bad Tolz, Germany) (Korotkoff I and V) with a cuff of appropriate size. Blood pressure has been always measured in the morning before daily drug intake (i.e, at trough 22-24 h after dosing) and after the subject has rested 10 min in a quiet room. Three successive BP readings were obtained at 1-min intervals and averaged.

Heart rate was measured by pulse palpation for 30 s, just before the BP measurements.

Body weight was measured with light clothes and without shoes and BMI was calculated as the weight in Kg divided by height in m squared.

Glycated hemoglobin level was measured by a high performance liquid chromatography method (DIAMAT, Bio-Rad, USA; normal values 4.2-6.2 %), with intra- and interassay coefficients of variation (CsV) of < 2 % [17].

Plasma glucose was assayed by glucose-oxidase method (GOD/PAP, Roche Diagnostics, Mannheim, Germany) with intra- and interassay CsV of < 2 % [18].

High sensitivity C-reactive protein was measured with use of latex-enhanced immunonephelometric assays on a BN II analyser (Dade Behring, Newark, Delaware, USA). The intra- and interassay CsV were 5.7 % and 1.3 %, respectively [19].

Tumor necrosis factor-α level was assessed using commercially available ELISA kits according to manufacturer’s instructions (Titer-Zyme EIA kit; Assay Designs, Ann Arbor, MI). Intraassay CsV were 4.5 % for low- and 3.6 % for high-concentration samples, whereas the interassay CsV were 6.0 % for low and 11.8 % for high-concentration samples, respectively [20].

Metalloproteinase-2, and MMP-9 levels were determined by a two-site ELISA methods using commercial reagents (Amersham Biosciences, Uppsala, Sweden). The intra- and interassay CsV for measuring MMP-2 levels were 5.4 %, and 8.3 %, respectively [21]. The intra- and interassay CsV to evaluate MMP-9 levels were 4.9 %, and 8.6 % [22].

Soluble intercellular adhesion molecule-1 and sVCAM-1 were assessed using commercially available ELISA kits according to manufacturer instructions (R & D Systems, Minneapolis, MN, USA). The intra- and interassay CsV were < 10 %, respectively [23, 24].

The level of isoprostanes in serum was determined by commercially available ELISA kit (Cayman Chemicals, Ann Arbor, Mich) [25].

Paraoxonase 1 activity in serum was measured using paraoxon as a substrate in the presence of 2 mM Ca^+2^ in 100 mM Tris-HCL buffer (pH = 8.0) [26].

### Ambulatory blood pressure monitoring

Twenty-four-hour BP and HR were evaluated by non-invasive automatic monitoring Spacelabs model 90207: Redmond, WA, USA) [27]. The interval between two subsequent measurements was 20 min from 07:00 to 23:00 h, and 30 min from 23:00 to 07:00 h, with a total number of about 64 measurements. Subjects were instructed to remain motionless and to record their activity on a diary sheet. The recorder automatically discarded false readings (e.g. arm in motion or sound interference during recording). Furthermore, additional readings were rejected during computer analysis if differential BP was < 20 mmHg, DBP was < 50 mmHg or SBP was > 260 mmHg in isolated readings. Less than 15 % of the total readings were rejected as artefacts. Recordings were only included in the study if at least 85 % of the maximal number of 64 readings during the 24-h period passed the deletion criteria. We calculated the following values from the 24-h BP profiles: mean 24-h SBP and DBP values, mean daytime SBP and DBP values, mean night-time SBP and DBP values, and the absolute difference between mean daytime and night-time SBP and mean DBP values.

### Statistical analysis

Data are expressed as mean ± standard deviation (SD). The statistical analysis of the data was performed by the statistical analysis software (SAS) system, version 6.12 (SAS Institute, Inc., Cary, NC, USA). The differences between the two groups in baseline characteristics were analyzed by the two-tailed Student’s *t*-test. Comparisons within and between groups were assessed by a mixed ANOVA. Differences between baseline and after 6-months’ treatment in each group in BP and oxidative stress parameters were analyzed with the Wilcoxon signed rank test. Comparisons of changes in BP and oxidative stress parameters between the two groups were performed with the Mann-Whitney *U*-test [28]; we adjusted results for potential confounding factors including SBP. Findings of *p* < 0.05 were considered significant. Considering as clinically significant a difference of at least 10 % compared with the baseline and an alpha error of 0.05, the actual sample size was adequate to obtain a power higher than 0.80 for all measured variables.

## Results

### Study sample

**Fig. 1:**
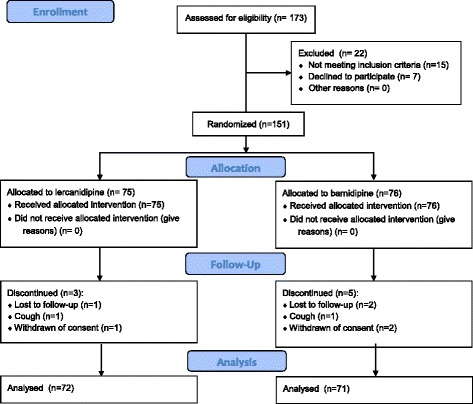
CONSORT 2010 Flow Diagram

We enrolled 151 patients; 75 were randomised to lercanidipine and 76 to barnidipine (Table 1). One hundred and forty-three patients completed the study. Eight patients did not complete the study and the reasons for prematurely withdrawal included: lost to follow-up (3 patients), cough (2 patients), withdrawn of consent (3 patients) (Fig. 1).

### Blood pressure and ABPM

Both barnidipine and lercanidipine resulted in a significant reduction in SBP and DBP (*p* < 0.001 vs baseline for barnidipine + losartan and *p* < 0.01 for lercanidipine + losartan), even if the BP reduction obtained with barnidipine + losartan was greater than that obtained with lercanidipine + losartan (*p* < 0.05). Data recorded with ABPM showed a similar trend, even if no differences were recorded in the descent of the nocturnal blood pressure of barnidipine and lercanidipine (Table 2).

**Table 2:** Effects of the two treatments on ambulatory blood pressure monitoring

	Losartan+barnidipine		Losartan+lercanidipine		
	Baseline	End of study	Baseline	End of study	*p* for interaction between groups
N	75	72	76	71	-
Sex (male/female)	37/38	36/36	36/40	35/36	ns
SBP (mmHg)	150.8 ± 9.7	130.2 ± 5.6**	152.5 ± 10.6	141.9 ± 6.5*	0.041
DBP (mmHg)	96.6 ± 5.4	84.5 ± 5.7**	96.7 ± 5.5	90.4 ± 5.9*	0.039
HR (beats/min)	70.3 ± 7.7	70.1 ± 7.6	72.6 ± 8.1	72.2 ± 7.9	ns
24 h SBP (mmHg)	142.5 ± 10.5	128.8 ± 7.3**	143.2 ± 10.8	134.9 ± 8.5*	0.042
24 h DBP (mmHg)	94.7 ± 7.9	84.5 ± 6.1**	94.3 ± 7.8	89 ± 7.1*	0.045
24 h MBP (mmHg)	118.2 ± 8.0	105.1 ± 6.1*	116.3 ± 7.6	110.3 ± 7.1	ns

Data are means ± SD

*SBP* systolic blood pressure, *DBP* diastolic blood pressure, *MBP* mean blood pressure, *HR* heart rate

*p < 0.01 vs baseline; **p < 0.001 vs baseline

### Oxidative stress parameters

Barnidipine + losartan reduced the levels of Hs-CRP and TNF-α (*p* < 0.05 vs baseline and vs lercanidipine + losartan). There were no significant differences between the two treatments on the levels of MMP-2 and -9. Barnidipine + losartan significantly reduced the levels of sVCAM-1 and sICAM-1 after 6 months of treatment, both compared to baseline and to lercanidipine + losartan (*p* < 0.05 for both). The levels of isoprostanes were reduced by barnidipine + losartan (*p* < 0.05 vs baseline and vs lercanidipine + losartan), while the levels of PON-1 remained unchanged with both treatments (Table 3).

**Table 3:** Effects of the two treatments on oxidative stress parameters

	Losartan+barnidipine		Losartan+lercanidipine		
	Baseline	End of study	Baseline	End of study	*p* for interaction between groups
N	75	72	76	71	-
Sex (male/female)	37/38	36/36	36/40	35/36	ns
BMI (Kg/m^2^)	28.5 ± 1.3	28.4 ± 1.2	28.2 ± 1.1	28.1 ± 1.0	ns
Fasting plasma glucose (mg/dl)	129.6 ± 9.7	125.8 ± 9.1	130.1 ± 10.2	127.6 ± 9.9	ns
HbA_1c_ (%)	7.0 ± 0.5	6.9 ± 0.4	6.9 ± 0.4	7.0 ± 0.5	ns
Hs-CRP (mg/l)	1.7 ± 0.9	1.2 ± 0.4*	1.6 ±0.8	1.5±0.6	0.047
TNF-α (ng/ml)	2.9 ± 1.7	2.2 ± 1.3*	2.8 ± 1.6	2.6 ± 1.4	0.042
MMP-2 (ng/ml)	1216.5 ± 124.1	965.1 ± 102.6*	1218.8 ± 126.8	978.3 ± 106.2*	ns
MMP-9 (ng/ml)	465.1 ± 50.1	382.5 ± 46.3*	462.2 ± 49.4	394.1 ± 48.5*	ns
sVCAM-1 (ng/ml)	637.8 ± 172.5	536.1 ± 112.7*	638.4 ± 173.1	592.7 ± 141.6	0.041
sICAM-1 (ng/ml)	201.5 ± 12.8	174.2 ± 8.1*	204.2 ± 13.3	198.6 ± 11.2	0.046
Isoprostanes (pg/ml)	114.3 ± 39.2	92.5 ± 28.5*	110.2 ± 36.8	103.4 ± 33.2	0.047
PON-1 (U/l)	160.5 ± 85.2	182.5 ± 99.2	157.1 ± 83.4	177.2 ± 95.7	ns

Data are means ± SD

*p < 0.05 vs baseline

*BMI* body mass index, *FPI* fasting plasma insulin, *HbA*_*1c*_ glycated hemoglobin, *Hs-CRP* high-sensitivity C-reactive protein, *TNF-α* tumor necrosis factor-α, *MMP-2* metalloproteinase-2, *MMP-9* metalloproteinase-9, *sVCAM-1* soluble vascular adhesion protein-1, *sICAM-1* soluble intercellular adhesion protein-1, *PON-1* paraoxonase-1

## Discussion

Barnidipine, and lercanidipine are both third-generation CCBs indicated for the treatment of hypertension [11]. In our study they both reduced BP control, with a better effect of barnidipine compared to lercanidipine, both in isolated BP measurements and during ABPM. This is in contrast with what we previously reported [29], where no differences were recorded between barnidipine and lercanidipine in reducing blood pressure. This can be due to the different population enrolled, diabetic hypertensive patients with left ventricular hypertrophy in the first study and diabetic hypertensive patients in the current trial. Regarding pleiotropic effects of these drugs, barnidipine is a new dihydropyridinic calcium antagonist with long-lasting vasodilator properties [30] in the absence of sympathetic activation [31], possible anti-oxidant effects [25], a slight beneficial effect on lipid profile [32], and a good effect on insulin sensitivity [33]. Also lercanidipine seems to give a reduction of white blood cells and peripheral polymorphonuclear leukocytes counts, of peripheral polymorphonuclear leukocytes apoptosis, and of C-reactive protein in hypertensive patients [34]. In our study, only barnidipine gave a decrease of Hs-CRP, TNF-α, sVCAM-1 and sICAM-1, both compared to baseline and lercanidipine. The better effect of barnidipine on endothelial damage markers compared to lercanidipine can be only partially explained by the greater BP reduction, because, after adjusting results for potential confounding factors including SBP, it does not seem to influence the results. This seems to suggest an intrinsic effect of the drug on endothelial damage markers.

On the other hand, isoprostanes are prostaglandin-like compounds formed in vivo from the free radical-catalyzed peroxidation of essential fatty acids, primarily arachidonic acid, without the direct action of cyclooxygenase enzymes. Isoprostane levels represent reliable markers of oxidative stress. Barnidipine administration caused a significant reduction of isoprostane levels, in line with what already reported by Spirou et al. [25].

Paraoxonase-1, instead, is synthesized in the liver and transported along with HDL in the plasma. It functions as an anti-oxidant; it prevents the oxidation of LDL. Its serum concentration is influenced by inflammatory changes and the levels of serum oxidised-LDL. In our study we did not record an effect of anti-hypertensive drug on this parameter.

We have already compared barnidipine and lercanidipine effects on echocardiographic parameters [29], however, this is, at our knowledge, the first study to directly compare the effects of two different CCBs on inflammation. Of course our study has some limitations, as the short study duration; moreover we did not assess estimated glomerular filtration rate variation during the study, and evaluated only some inflammatory markers, focusing our attention on a few of them.

## Conclusions

In addition to giving a greater reduction of BP, barnidipine + losartan gave an improvement of some parameters indicative of endothelial damage and oxidative stress in diabetic and hypertensive patients.

### Availability of data and materials

Our data consist of clinical datasets. Given that clinical datasets publication would not guarantee participants’ rights to privacy, we prefer to not share our data. In the informed consent, patients agreed to share their personal data only for medical reasons; publication of single data was not contemplated in the informed consent.

## Abbreviations

BMI: body mass index
FPI: fasting plasma insulin
HbA_1c_: glycated hemoglobin
Hs-CRP: high-sensitivity C-reactive protein
MMP-2: metalloproteinase-2
MMP-9: metalloproteinase-9
PON-1: paraoxonase-1
sICAM-1: soluble intercellular adhesion protein-1
sVCAM-1: soluble vascular adhesion protein-1
TNF-α: tumor necrosis factor-α
